# A review of the current status and progress in difficult airway assessment research

**DOI:** 10.1186/s40001-024-01759-x

**Published:** 2024-03-13

**Authors:** Haoming Chen, Yuqi Zheng, Qiang Fu, Peng Li

**Affiliations:** 1 Department of Anesthesiology, Sichuan Academy of Medical Sciences and Sichuan Provincial People’s Hospital, University of Electronic Science and Technology of China, Chengdu, China; 2 Southwest Medical University, Luzhou, China; 3 Department of Anesthesiology, The Third People’s Hospital of Chengdu, Chengdu, China; 4 Department of Anesthesiology, The First People’s Hospital of Guangyuan, Guangyuan, China

**Keywords:** Difficult airway, Difficult intubation, Evaluation, Artificial intelligence, Machine learning, Deep learning, Application

## Abstract

A difficult airway is a situation in which an anesthesiologist with more than 5 years of experience encounters difficulty with intubation or mask ventilation. According to the *2022 American Society of Anesthesiologists Practice Guidelines for the Management of Difficult Airway*, difficult airways are subdivided into seven detailed categories. This condition can lead to serious adverse events and therefore must be diagnosed accurately and quickly. In this review, we comprehensively summarize and discuss the different methods used in clinical practice and research to assess difficult airways, including medical history, simple bedside assessment, comprehensive assessment of indicators, preoperative endoscopic airway examination, imaging, computer-assisted airway reconstruction, and 3D-printing techniques. We also discuss in detail the latest trends in difficult airway assessment through mathematical methods and artificial intelligence. With the continuous development of artificial intelligence and other technologies, in the near future, we will be able to predict whether a patient has a difficult airway simply by taking an image of the patient's face through a cell phone program. Artificial intelligence and other technologies will bring great changes to the development of airway assessment, and at the same time raise some new questions that we should think about.

**Supplementary Information:**

The online version contains supplementary material available at 10.1186/s40001-024-01759-x.

## Introduction

A “difficult airway” is conventionally defined as a clinical situation where a trained anesthesiologist with more than 5 years’ experience has difficulty with facemask ventilation or intubation of an artificial airway [1]. According to the *2022 American Society of Anesthesiologists Practice Guideline for Management of the Difficult Airway*, difficult airways can be further divided into seven types: difficult facemask ventilation, difficult laryngoscope, difficult supraglottic airway ventilation, difficult or failed tracheal intubation, difficult or failed tracheal extubation, difficult or failed invasive airway, and inadequate ventilation [2]. Despite the tremendous advances in anesthesia techniques and equipment, the occurrence of a difficult airway during intubation still leads to serious anesthesia-related injuries and is the most common cause of anesthesia malpractice claims [3]. The worst case situation is "failure to intubate and ventilate", and up to one-third of anesthesia-related deaths are due to failure to intubate and ventilate, so this deserves our utmost attention. The occurrence of a difficult airway can lead to tracheal or esophageal injury, aspiration, and severe hypoxemia, which can cause irreversible brain damage and lead to death [4, 5]. Therefore, accurate perioperative assessment can significantly reduce the incidence of perioperative adverse events [6]. Unfortunately, there is still no consensus on the best method to assess a difficult airway [7, 8]. We will discuss below some traditional and more established high-end methods of difficult airway assessment and compare some advantages and disadvantages of each of them.

Artificial Intelligence (AI), an emerging discipline, has been in existence for just a century, but it has already impacted a wide range of industries, including, of course, the healthcare industry. The potential of AI in healthcare is enormous [9]. At the same time, the birth and rapid development of technologies such as face recognition and analysis have made it possible to apply them to predict difficult airways. We will detail the current state of research and applications of AI in predicting difficult airways in this review.

## Traditional methods of difficult airway assessment

The patient's medical history is one of the important pieces of information when evaluating a difficult airway. Certain conditions have been shown to be strongly associated with difficult airways, such as congenital disorders that alter the face or mouth, rheumatoid arthritis, acromegaly, a history of head and neck radiation therapy, and obstructive sleep apnea syndrome [10–12].

Current research suggests that a patient's previous difficult airway diagnosis is the most meaningful warning factor for the next airway management physician who sees that patient [13]. Therefore, we strongly recommend that countries establish a database of patients with difficult airways [14]. The database would store in detail the patient's accurate airway data as well as the last physician's management (similar to an infectious disease control system). Some European and North American countries have already established difficult airway databases. They also use special visual warning signs such as wristbands for hospitalized patients who have had a difficult airway diagnosis to better alert physicians [15]. However, difficult airway databases have not yet been established in most parts of the world.

Another way to traditionally diagnose a difficult airway is a simple bedside assessment. The physician assesses the patient's facial and mandibular features such as mouth opening, buck teeth, modified Mallampati classification, and the upper lip bite test (ULBT) [16]. The physician also performs some simple anatomical measurements, including hyomental distance, sternomental distance, inter-incisor gap and neck circumference (Additional file 1: Table S1) [17–19]. The two main problems with bedside testing used to diagnose the presence of a difficult airway are the setting of cutoff values and the difference in cutoff values between different subgroups, respectively. The cutoff values for these tests may vary considerably between age groups or by gender and ethnicity, so clinicians need to select appropriate screening indices based on patient and region.

Due to the subjectivity and poor accuracy of using a single factor to predict a difficult airway, comprehensive assessment algorithms have been created, such as the Wilson score, the SARI score, and the modified LEMON score (Additional file 1: Table S2) [19, 20]. By using multiple predictors, large variations due to assessor subjectivity will be minimized, thus improving the accuracy of difficult airway prediction. However, these comprehensive assessment tools are complex and time-consuming, making them difficult to apply in daily practice. More research is available to simplify and improve the relevant parameters [21].

## Established high-end methods of difficult airway assessment

### Preoperative endoscopic airway examination (PEAE)

Various visible-light endoscopes, such as the video laryngoscope, visual light sticks, transnasal flexible endoscopic laryngoscope and fiberoptic bronchoscope, can help anesthesiologists view airway anatomy directly [22, 23]. The use of these endoscopes can dramatically reduce the rate of failed intubation and laryngeal/airway trauma [24]. PEAE of suspected difficult airway can allow detailed assessment to guide appropriate preoperative preparation [25]. In some extremely rare or exceptional cases, preoperative use of visualization endoscopy to observe the alignment and stenosis of the patient's airway is necessary to minimize some catastrophic complications and failure to ventilate and intubate [26]. At the same time, the development of awake intubation techniques with some visualization endoscopes has made it possible to solve the majority of difficult airways. However, the endoscopes are expensive, the corresponding examinations are time-consuming, and patients’ experience is painful, also only some anesthesiologists can operate them, which explains why the use of endoscopes will be restricted [27].

### Imaging examinations

X-ray, computed tomography (CT), magnetic resonance imaging (MRI) and ultrasonography have been widely used to diagnose difficult airway [28]. X-ray imaging can clearly visualize skeletal structures, and it has led to the proposal of different distances between anatomical points as diagnostic markers of the condition. Examples include linear distance from the interior border of the mandible to the hyoid bone, which can predict tongue size, and maximum retropalatal area in the axial view, which can predict the pharyngeal space [29, 30]. CT and MRI can provide detailed information about factors that can lead to difficult airway, including anatomical location of tumors, involvement of secondary structures, and distortion or narrowing of the airway [31]. Some of the problems we can't ignore with X-rays, CT and MRI are their radiation, inconvenience and high cost, but not with ultrasound. Ultrasonography can image not only some anatomical structures also visible by video laryngoscopy, such as the tongue, epiglottis, and glottis, but also some anatomical structures that are not visible with laryngoscopy, such as the hyoid bone, cricoid cartilage, and soft tissues of the neck [32, 33]. In fact, the diagnostic index and AUC of ultrasound were similar to those of CT and X-ray in predicting difficult airways, and the diagnostic value of all three was much better than the modified Mallampati score [34]. This fact and its relative ease of use, safety, widespread availability, low cost and reproducibility argue for using it as a routine tool for diagnosis of difficult airway. This may become easier with the development of pocket-sized ultrasound devices [35]. By simply carrying a laptop-sized ultrasound instrument with us, we can quickly perform a rapid assessment of the patient's airway anatomy at the bedside, which is very useful for patients who have failed intubation but are successfully ventilated and need emergency surgery.

### Computer-aided airway reconstruction and three-dimensional (3D) printing techniques

Computer modelling is increasingly explored as a way to understand difficult airway and develop adaptive strategies by reconstructing 3D airway models from two-dimensional images and related data [36, 37]. This approach can precisely model anatomical structures and the biomechanics of intubation [38]. For some extremely rare diseases, 3D printing of such models can help anesthesiologists formulate the safest possible plan to manage difficult airway, and it can facilitate the development of new intubation devices [39, 40]. In patients with head and neck cancer, for example, where tumors in the oropharynx, larynx and hypopharynx severely distort and narrow the anatomy of the airway, surgeons and anesthesiologists use 3D augmented reality software combined with 3D printed modeling technology to perform a preoperative airway assessment, which allows the anesthesiologist and surgeon to anticipate all critical steps and adjust the intubation plan accordingly. These technologies are expensive, but their value is far greater than their cost in rare and special cases and post-operative case studies, but the high end and expensive equipment required limits the spread of this technology.

## Emerging novel methods for difficult airway assessment

### Create predictive models through mathematical methods

The creation of predictive models through mathematical methods is a standardized set of processes that use mathematical equations to explore the patterns of change in variables based on data. In the previous decades, statistical modeling was very popular in the medical community for prediction of survival outcomes, diagnosis of diseases, and epidemiological trend prediction of infectious diseases, etc. [41, 42]. The main statistical methods include logistic regression, logistic LASSO regression, cox regression, etc. [43]. The choice of statistical methods to build predictive models is determined by the type and number of dependent and independent variables. The common prediction models currently available are mathematical equations, nomogram, etc. [44]. The general process of creating prediction models by mathematical methods is currently conventional as follows: first, some indicators that may be related to the outcome variables are selected based on clinical experience, then statistically significant indicators are screened out by some statistical methods (e.g. single-factor logistic regression, multi-factor logistic regression, etc.) to build prediction models, and finally the accuracy of the prediction models will be evaluated by using evaluation indicators such as ROC curves to assess whether further optimization is needed. There have also been many studies on creating prediction models for difficult airways through mathematical methods. For example, Bin Wang et al. created a mathematical nomogram for difficult airway prediction using some anatomical indicators by ultrasound measurements and some factors related to difficult airway such as age [45]. The prediction model created by mathematical methods does provide a great aid in the determination of outcome variables, but the process of implementation is still not very satisfactory from our point of view. First, the amount of data handled by mathematical methods to create prediction models is relatively small, secondly, the researcher must understand how the data were collected, the statistical characteristics of the estimates (including p-values and unbiased estimates), the potential distribution patterns of the population under study, and other processes, and most importantly, the researcher must propose parameters with predictive power by themselves, which may result in the neglect of some indicators that may have predictive significance. Although the creation of predictive models through mathematical methods suffers from some of the problems mentioned above, we can't completely deny the significance of their existence, and the statistical methods used in this are also the prototype of the methods used by the artificial intelligence that will be born later on, but the artificial intelligence makes up for some of the shortcomings mentioned above very well.

### AI in difficult airway assessment

#### AI

Since the concept of AI was first introduced in 1956, its theory and technology have continued to mature, and the changes it has caused have swept through all walks of life like a tsunami. AI is a technical science that studies and develops theories, methods, technologies and application systems for simulating, extending and expanding human intelligence, and its common methods include expert systems, machine learning, deep learning, natural language processing, computer vision, evolutionary algorithms, and knowledge representation and reasoning [46]. AI have been widely adopted in medicine, including in anesthesiology [47]. For example, AI algorithms have been used to predict in-hospital mortality rate based on intraoperative characteristics and predict hypotension before it occurs during surgery, and all have high prediction accuracy [48, 49].

A part of research has been published on AI and difficult airway assessment, in which the main AI methods used are machine learning, deep learning and computer vision. There are two main directions in which existing research has been applied in predicting difficult airway through AI methods. The first is to manually collect characteristic factors that may be associated with a difficult airway and then train them using AI algorithms with a view to identify the characteristic factor that best predicts a difficult airway, to predict the risk of a difficult airway occurring based on this characteristic factor, or to combine the top-ranked predictive accuracy of the characteristic factors into a single model for predicting a difficult airway. For example, in a study on predicting difficult airways for thyroid surgery, the authors used 10 AI algorithms trained on labeled input features, ultimately concluding that age, gender, weight, height, and body mass index were the five most important factors in identifying difficult airways. However, this method of predicting difficult airways is still semi-automated and requires us to collect, extract and input data, not quite the same as the fully automated analysis we originally envisioned [50]. Another direction to predict difficult airways through AI is based on the digitization of artificial intelligence and the number and availability of medical images as a source of data [51]. Our approach is to collect images of the patient's face in all directions, at different mouth openings and inside the mouth, and combine them with techniques such as facial recognition, which leads to automated algorithm-based AI predictions and interpretations. This approach does not require us to provide training data with labels in advance, and is the one that we are most interested in actually applying in the clinic. Some current research suggests that AI is already relative to general radiologists and pathologists in recognizing the imaging presentation of some diseases, but it takes less time, giving us more confidence in predicting difficult airways through complete automation of AI [52, 53].

#### Machine learning

**Fig. 1:**
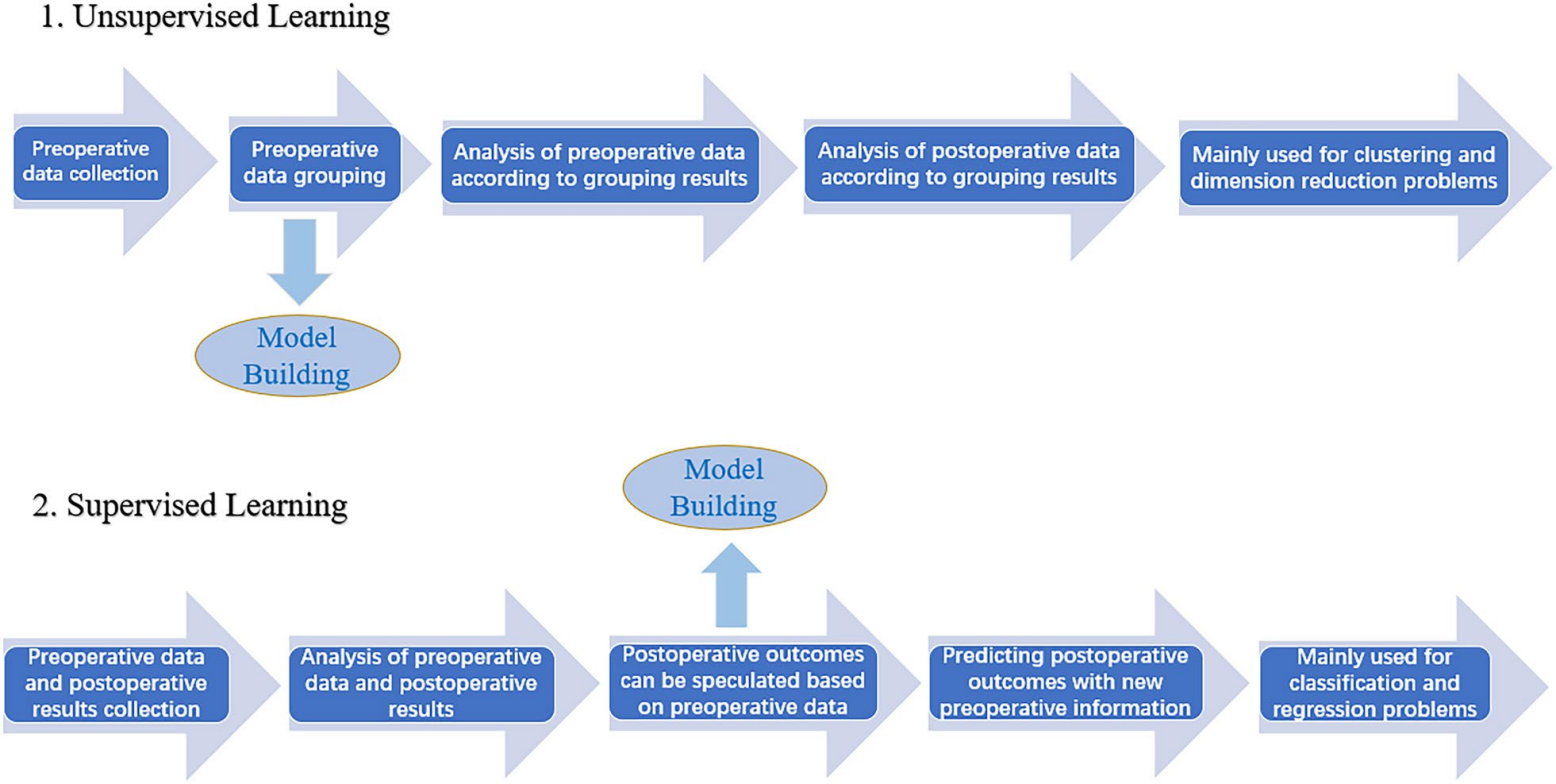
Explanation and difference between unsupervised and supervised learning

Machine learning, the core of AI, is the fundamental way to make computers intelligent. Machine learning simply means training a model by inputting a large amount of training data, so that the model can grasp the potential rules contained in the data, and then make accurate classification or prediction for the new input data. Machine learning models can be divided into supervised learning, unsupervised learning, semi-supervised learning and reinforcement learning, and the two main methods of machine model learning are supervised learning and unsupervised learning, which are widely used in medicine [54]. If we want to speculate on postoperative outcomes by collecting preoperative data, the general interpretation and difference between these two models is shown in Fig. 1. Algorithms for machine models include random forests, support vector machines, multilayer perceptron, gradient boosting and Bayesian algorithms [55]. There have been many studies that have successfully implemented fully automated difficult airway prediction through machine learning methods. For example, Cuendet et al. were the first to combine a random forest approach to machine learning with facial recognition techniques to enable fully automated difficult airway prediction by taking images of a patient's face [56]. In machine learning, one of the most important processes for automatic identification of difficult airways through facial images is the selection and extraction of facial features, which is where the differences between each model lie. Compared to creating predictive models by machine learning and by mathematical methods, machine learning focuses on exploring the relationships and structures exhibited by the data, is more concerned with the predictive power of the model, and handles a greater breadth and depth of data, while by mathematical methods it focuses on evaluating the relationships and structures embodied in small samples of data to generalize them in the aggregate, is more concerned with the interpretability of the model, and handles a relatively smaller and narrower volume of data.

#### Deep learning

The birth of deep learning as a new research direction in the field of machine learning has brought machine learning closer to its original goal, AI. The ultimate goal of deep learning is to enable robots to have analytical learning capabilities like humans, capable of recognizing data such as text, images and sounds, and the main pathways can be divided into convolutional neural network, fully convolutional network, recurrent neural network and generative adversarial network [57, 58]. Its algorithms can be further divided into Keras, Tensorflow, Pytorch, Caffe and Theano [59].

**Fig. 2:**
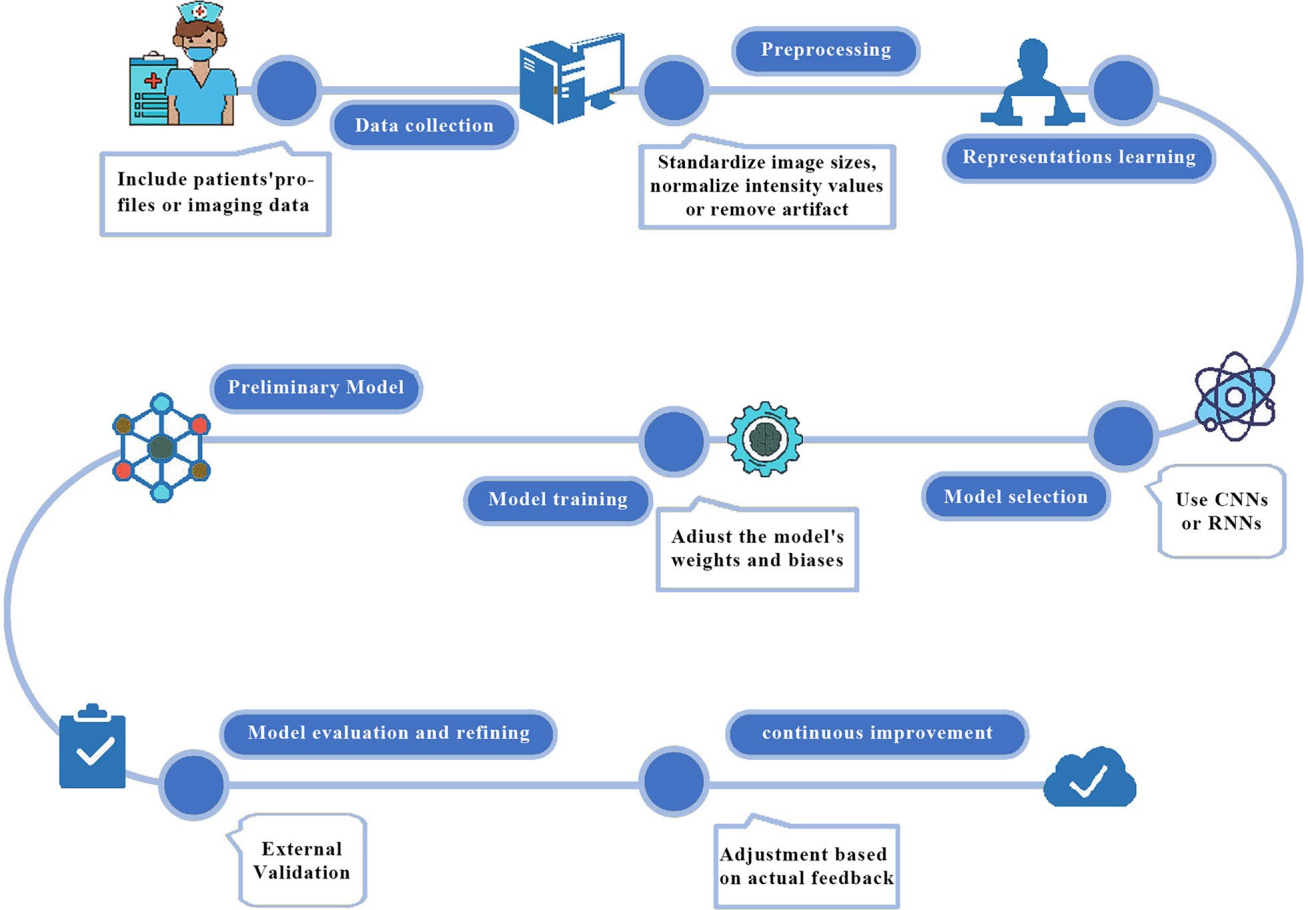
Flow diagram of creating model by deep learning

Deep learning, which analyzes and classifies new data by learning the intrinsic patterns and levels of representation of sample data, is now being applied to the prediction of difficult airways [60]. For example, in a Japanese study, the authors used a deep learning convolutional neural network approach, combined with class activation heat map techniques, to enable the recognition of difficult airways through AI recognition of facial profiles and thus the recognition of difficult airways [61]. The process of building a model for predicting a difficult airway through a deep learning approach can be summarized in Fig. 2.

In addition to the direct collection of patient's facial images for difficult airway prediction by deep learning, there are some studies that analyze the radiographic pictures of patient's head, face, and neck for difficult airway prediction by deep learning. For example H-Y CHO et al. developed a model for predicting a difficult airway based on convolutional neural network algorithm by using patient's cervical spine lateral X-ray images [62]. The emergence of these models and algorithms may provide a new way of thinking about the clinical assessment of difficult airways. However, deep learning models are based on a “black box” approach, which has led to questions about their lack of interpretability, which is one of the main barriers to the generalization of deep learning [63]. Some studies have used post-hoc methods or supervised machine learning models to explain the results, however, this interpretation of deep learning methods has been criticized by many academics who believe that it should not be necessary to explain how deep learning models work. More efforts are needed to improve the interpretability of deep learning algorithms while gaining wider acceptance.

#### Applications based on AI for predicting difficult airways

There are a number of apps available for predicting difficult airways, such as The Difficult Airway App, Airway Triage and DI DETECTION. Of these apps, we believe that Airway Triage and DI DETECTION are designed to come close to what we envision as "AI". Airway Triage (version 6.1, created by St Mobile Anesthesiology Service Holland) is supported by the *Airway Management Academy* and is intended for anesthesiologists, emergency and critical care physicians. The user selects the patient characteristics in the PHASE checklist screen, which are Patient, History, Airway, Surgical Procedure, and Evaluation of Vital Signs, and then the software categorizes the airway as basic or advanced based on the selected characteristics. If the airway is rated as advanced, the screen will jump to the HELPET checklist screen to evaluate the complexity factors, including Human Factors (applies to you or the team), Experience, Location of the Airway Procedure, Patient Factors, Equipment, Time pressure, and then the advanced airway is further categorized and graded as either low complexity or high complexity, with the interface roughly in Additional file 1: Figure S1. The app also has functions such as Circles of Life Approach, which is used to assess whether the user is capable of handling that airway or no. However, the app suffers from issues such as still requiring a lot of maneuvering by the user and being relatively cumbersome, but for beginners who are just learning about airway management, this app is relatively comprehensive and detailed. Another app called DI DETECTION (created by Khon Kaen University) is the closest app currently available to our ideal—that is, taking a photo of the patient's face, the app automatically determines if the patient has a difficult airway. The app starts by taking a photo of the patient's side face at the closest distance (must have epiglottis and thyroid cartilage in the picture), then manually selecting the midpoint of the patient's lips in the photo, then selecting the point of the patient's lower lip and the outermost point of the patient's chin, and finally selecting the point of the outermost point of the patient's thyroid cartilage, and the software automatically generates a determination of whether or not the patient has a difficult airway, as shown in the Additional file 1: Figure S2. The birth of this software, which gives the results of airway assessment by determining the distance between the patient's thyromental distance, is very innovative, but it still has the limitations of a single judgment indicator and the need to manually select the identification points. We expect that in the near future, more and better applications will be available for the determination of difficult airways by means of multiple indicators and simpler operation.

## Challenges of AI in predicting difficult airways

In this paper, we comprehensively summarize the existing traditional, high-end mature and emerging methods of difficult airway assessment. In actual clinical work, we should choose the most appropriate airway assessment method according to the actual situation of the patient and the assessor's own level of competence, and we give a flowchart of our recommended airway assessment algorithm (Additional file 1: Figure S3). Correct advanced difficult airway determination is beneficial in avoiding serious anesthetic complications. Given the low accuracy and time-consuming character of many current methods for diagnosing difficult airways, we recommend the use of AI algorithms based entirely on patient facial image data. Advanced AI algorithms such as face recognition can help improve the sensitivity and specificity of difficult airway diagnosis and provide a reliable reference for beginners in airway assessment.

However, the predictive algorithms and models for building difficult airways through AI are not perfect and are not without challenges and limitations. AI is based on data, and we cannot ignore the data ethics issues that come with big data. When we apply data such as patients' facial images, we must improve informed consent and strictly comply with relevant laws and regulations, and do a good job of data confidentiality and authorization, etc. to prevent data leakage and misuse [64]. Second, some difficult airway prediction models are currently over-fitted to improve model accuracy, resulting in poor extrapolation, while many models are built from small single-center data, and these single-center data may also be biased. To improve the generalization and robustness of model algorithms, we can do so by, for example, extending the training dataset, establishing comprehensive quality control and standardization tools, and using multi-institutional data sharing and validation [65]. In addition, the actual use of AI into clinical settings requires regulatory approval. In most countries, one of the criticalities that dominates whether approval can be passed is the interpretability of the software, and the lack of interpretability of AI can make it difficult to pass regulation [66]. However, the FDA in the United States has begun to approve some AI-based machines for clinical applications, which brings a glimmer of light to break through this regulatory barrier. Finally, the current studies related to difficult airway prediction are poorly written and reported with insufficient standardization, especially in the model development part, which makes it difficult for others to imitate and reproduce the results of their models [67]. The inability to imitate and reproduce the model prevents external validation of the developed model, leading to the birth of the dilemma that only the model is developed and it is difficult to actually apply the model to the clinic in a practical way. That is why we urgently need standardized written entries. So while there has been a lot of research into predicting difficult airways through AI, there are still significant technical, ethical, regulatory and administrative issues to overcome when applying it to actual clinical work.

Another major issue that exists with predicting difficult airways through AI is the objective quantification of difficult airway judgments. This poses many challenges to the accurate judgment of a difficult airway due to the subjectivity of the individual making the determination as well as a variety of other factors. However, we believe that even with such limitations, AI can achieve performance that is close to or even exceeds that of humans, so predicting difficult airways through AI is full of promise.

### Supplementary Information

**Additional file 1:** **Table S1.** Simple bedside assessment to differentiate difficult airway. **Table S2.** Comprehensive index assessment to differentiate difficult airway. **Figure S1.** The operation interface of Airway Triage (version 6.1, created by St Mobile Anesthesiology Service Holland). **Figure S2.**The operation interface of DI DETECTION (created by Khon Kaen University). **Figure S3.** Flow diagram of determining difficult airway in actual clinical work.

## Data Availability

Not applicable.

## Abbreviations

AI: Artificial intelligence
ULBT: Upper lip bite test
PEAE: Preoperative Endoscopic Airway Examination
CT: Computed tomography
MRI: Magnetic resonance imaging
3D: Three-dimensional
