# Supplementary material for: A review of the current status and progress in difficult airway assessment research
Source: Eur J Med Res. 2024 Mar 13;29:172. doi: 10.1186/s40001-024-01759-x (PMC10935786; doi:10.1186/s40001-024-01759-x)
Supplement: Supplementary file 1 — Additional file 1: Table S1. Simple bedside assessment to differentiate difficult airway. Table S2. Comprehensive index assessment to differentiate difficult airway. Figure S1. The operation interface of Airway Triage (version 6.1, created by St Mobile Anesthesiology Service Holland). Figure S2.The operation interface of DI DETECTION (created by Khon Kaen University). Figure S3. Flow diagram of determining difficult airway in actual clinical work. [file 40001_2024_1759_MOESM1_ESM.docx]

| Additional file 1: Table S1 Simple bedside assessment to differentiate difficult airway | | |
| --- | --- | --- |
| Evaluation Indicators | Normal | Abnormal |
| Degree of mouth opening | ≥5 cm | <5 cm |
| Head and neck movement | ≥90° | <90° |
| Dentition | Normal incisors or the total absence of teeth | Mobile or protruding incisors |
| Beard | None or little | bushy beard |
| The upper lip bite test | Class I, Class II, | Class III |
| Mallampati classification | Grade I and II | Grade III and IV |
| Modified Mallampati classification | Grade I and II | Grade III and IV |
| Thyromental distance | ≥6.0cm | <6.0 cm |
| Hyomental distance | ≥3.5cm | <3-3.5cm |
| Sternomental distance | ≥ 12.5 cm | <12.5 cm |
| Neck circumference | ≤ 42 cm | > 42 cm |
| Hyomental distance ratio (1) | > 1.2 | ≤ 1.2 |
| Ratio of height to thyromental distance (2) | ≤ 23.5 | > 23.5 |
| Ratio of height to sternomental distance (2) | ≤ 12.5 | > 12.5 |
| Body mass index | ≤ 30 | > 30 |
| Temporomandibular joint movement | move the lower incisors in front of the upper incisors | remain behind or aligned with the upper incisors |
| NEMA（Neck Circumference Minus Acromion-Acromion Distance）(3) | <5cm | ≥5cm |

| Additional file 1: Table S2 Comprehensive index assessment to differentiate difficult airway | | |
| --- | --- | --- |
| Name of the model | Evaluation Indicators | Judgment criteria |
| Wilson score (4) | 1. Weight (< 90 kg, 90 - 110 kg, > 110 kg) 2. The vertical head and neck movement (> 90°, = 90°, < 90°) 3. Jaw movement (IG > 5 cm and (or) Slux > 0，IG = 5 cm and (or) Slux = 0, IG < 5 cm and (or) SLux < 0) 4. Receding mandible (normal, moderate, severe) 5. Buck teeth (normal, moderate, severe) | 5 indicators were scored from 0 , 1 , 2 and when the total score ≥ 2, it indicated possible difficult intubation |
| SARI score (5) | 1.mouth opening 2. thyromental distance 3. Mallampati classification 4. neck movement 5. ability to prognath 6. weight 7. history of previous difficult intubation risk factors were assigned a score of 0, 1 or 2 depending on statistical significance | SARI score ≥ 4, it indicated possible difficult intubation |
| modified LEMON score (6) | 1. External looking: facial trauma → + 1; larger incisors → + 1; beard or moustache → + 1; large tongue → + 1 2. Evaluate the 3-3-2 rule: inter-incisor distance < 3 finger breadths → + 1; hyoid-to-mental distance < 3 finger breadths → + 1; thyroid-to-hyoid distance < 2 finger breadths → + 1 3. Obstruction: any conditions causing airway obstruction → + 1 4. Neck mobility: limited neck mobility or applying neck immobilizer → + 1 | Score ≥ 3, it indicated possible difficult intubation |
| The new Naguib clinical model (7) | Prediction score = 0.2262 - 0.4621 x TMD (cm) + 2.5516 x MM (class 1 or 2 → 0, class 3 or 4 → 1) - 1.1461 x MOD (cm) + 0.0433 x height (cm) | Score > 0, it indicated possible difficult intubation |
| Arne´ system (8) | 1. Previous knowledge of difficult intubation (No → 0, Yes → + 10) 2. Diseases associated with difficult intubation (No → 0, Yes → + 5) 3. Clinical symptoms of airway pathology (No → 0, Yes → + 3) 4. IG and mandible subluxation (IG ≥ 5 cm or SLux >0 → 0, 3.5 < IG < 5.0 cm and SLux = 0 → + 3, IG < 3.5 cm and SLux < 0 → + 13) 5. Thyromental distance(≥6.5 cm → 0, <6.5 cm → + 4) 6. Maximum range of head and neck movement (More than 100° → 0, About 90° (± 10°) → + 2, Less than 80° → + 5) 7. Mallampati score (Class 1 → 0, Class2 → + 2, Class 3 → + 6, Class 4 → + 8) | Score > 11, it indicated possible difficult intubation |
| HEAVEN criteria (9) | 1. H - Hypoxemia 2. E - Extremes of size 3. A - Anatomic challenge 4. V - Vomit/blood/fluid 5. E - Exsanguination 6. N - Neck mobility issues | For each item present, the intubation success rate decreases |
| STOP-Bang questionnaire (10) | 1. Snoring – Do you snore loudly (louder than talking or loud enough to be heard through closed doors)? 2. Tired – Do you often feel tired, fatigued, or sleepy during daytime? 3. Observed – Has anyone observed you stop breathing during your sleep? 4. Blood Pressure – Do you have or are you being treated for high blood pressure? 5. BMI – More than 35 kg/㎡? 6. Age – Over 50 year old? 7. Neck circumference – Greater than 40 cm? 8. Gender – Gender male? | Patients with STOP-Bang scores ≥3 are more likely to have difficult intubation |
| El-Ganzouri score (7) | 1. MOD (mm): ≥ 40 → 0, < 40 → + 1 2.TMD (mm): > 65 → 0, 60 - 65 → + 1, < 60 → + 2 3.Modified Mallampati: Grade I → 0, Grade II → + 1，Grade III → + 2，Grade IV → + 2 4.HNM (degrees): > 90 → 0, 80 - 90 → + 1, <80 → + 2 5. Ability to prognath: yes → 0, no → + 1 6. Body weight (kg): < 90 → 0, 90 - 110 → + 1, >110 → + 2 7. History of difficult intubation: none → 0, questionable → + 1, definite → + 2 | A score ≥ 4 was considered positive for difficult intubation |
| Langeron clinical score (7) | 1. BMI: < 25 → 0, 25 - 34 → 0, ≥ 35 → + 1 2. MM: class I → 0, class II → + 2, class III → + 4, class IV → + 6 3. MOD (mm): > 50 → 0, 36 - 50 → + 1, ≤ 35 → + 2 4. TMD (mm): ≥ 90 → 0, 61 - 89 → + 1, ≤ 60 → + 2 5. Sex: woman → 0, man → + 1 6. Receding mandible: no → 0, yes → + 2 | a score ≥ 5 was considered positive for difficult intubation |
| Barcelona score(Surgery for laryngopharyngeal disorders only）(11) | 1. Mallampati III - IV 2. Thyromental distance < 6.5 cm 3. Mouth opening < 4 cm 4. Limitation temporomandibular joint 5. Pathological dentition 6. Maxillary deficiency 7. Neck mobility < 90° 8. Receding mandible 9. BMI > 30 10. Symptoms laryngeal dysfunction 11. Tumour supraglottic region | Good predictive ability for difficult intubation when ≥ 5 indicators |
| KG = Kilogram; IG = inter-incisor gap; SLux = subluxation (maximal forward protrusion of the lower incisors beyond the upper incisors); MOD = mouth opening distance; SD = thyrosternal distance; NC = neck circumference; TMD = thyromental distance; HNM = head and neck movement; MM= Modified Mallampati; BMI = body mass index | | |


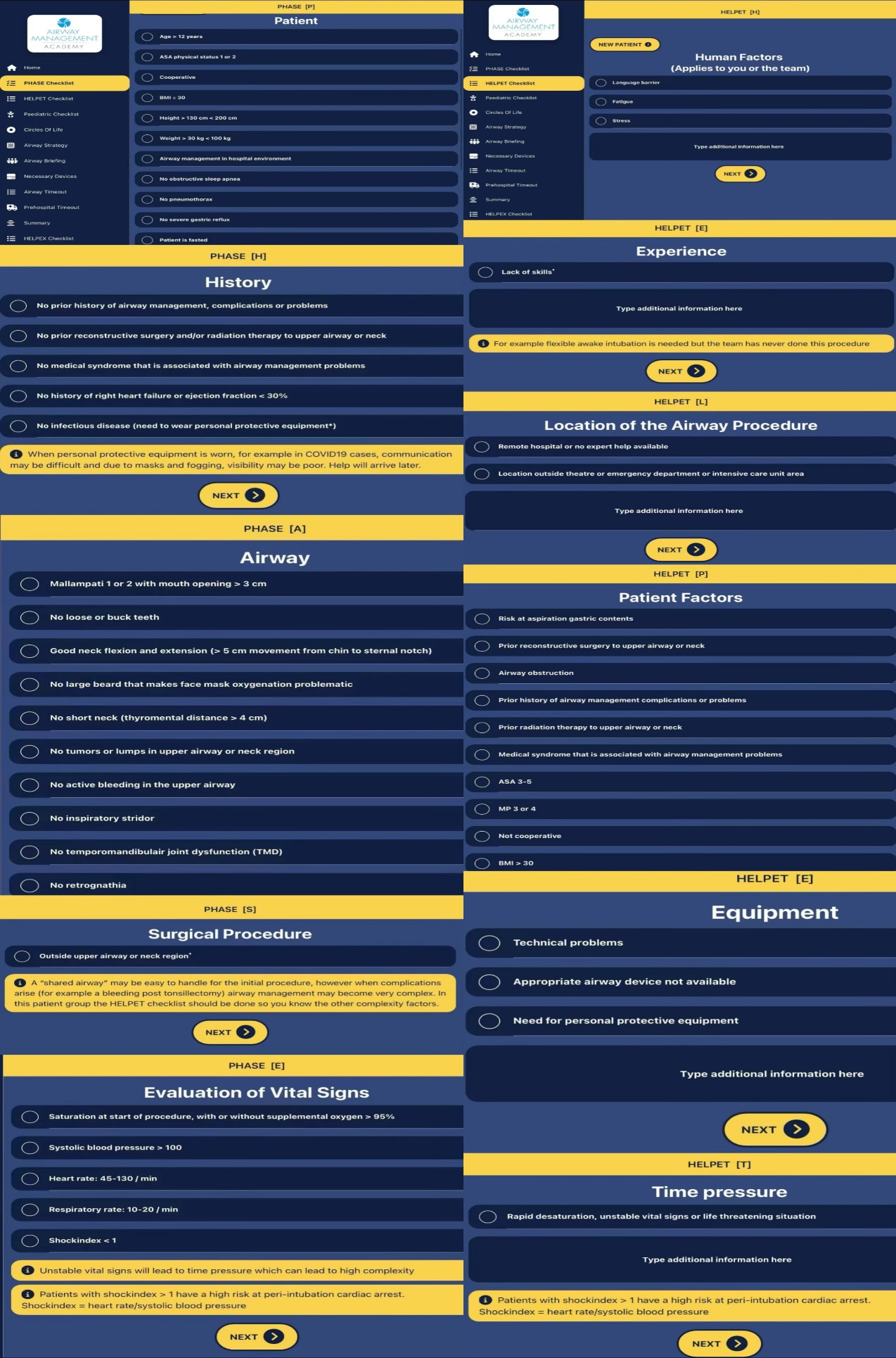


Additional file 1: Figure S1 The operation interface of Airway Triage (version 6.1, created by St Mobile Anesthesiology Service Holland)


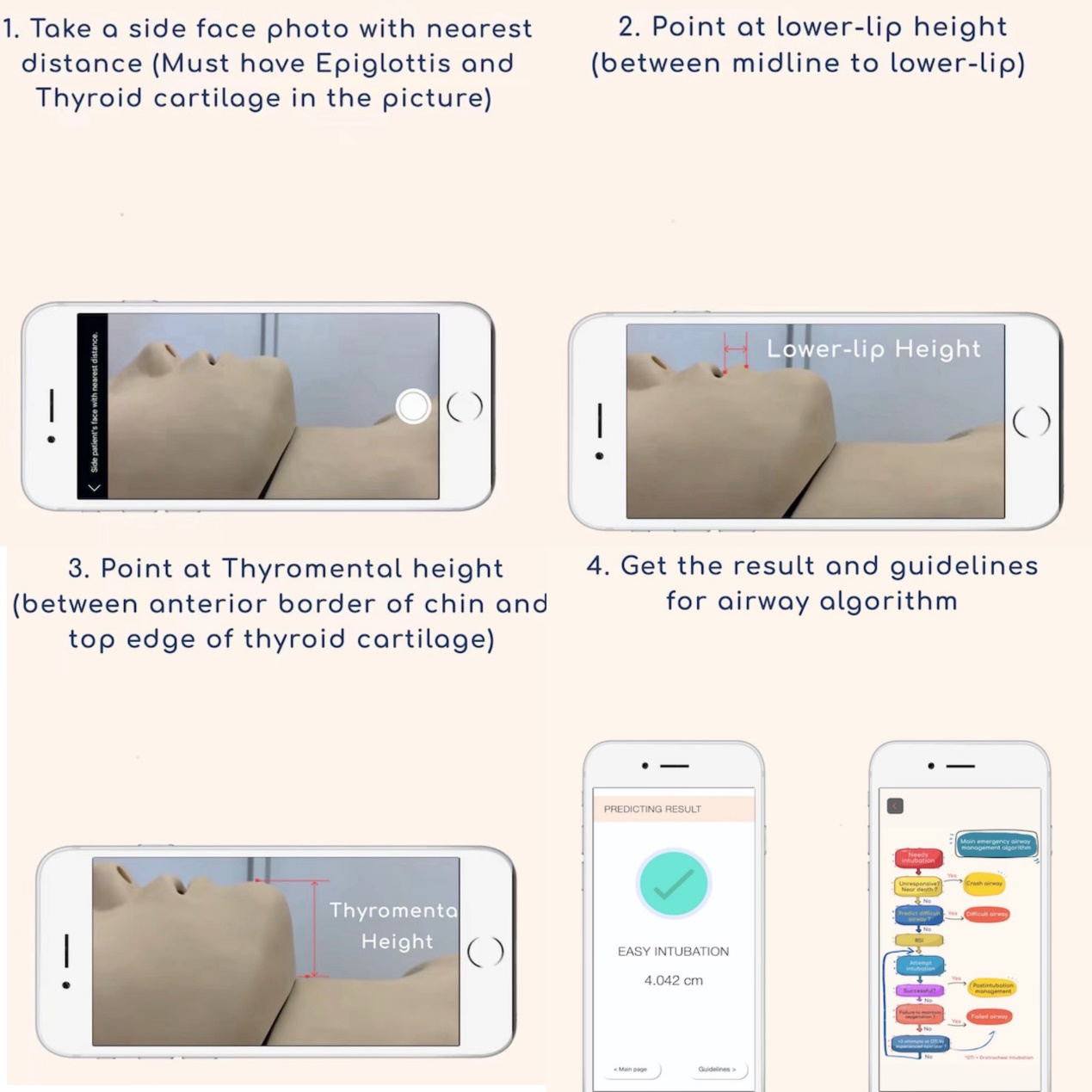


Additional file 1: Figure S2 The operation interface of DI DETECTION (created by Khon Kaen University)


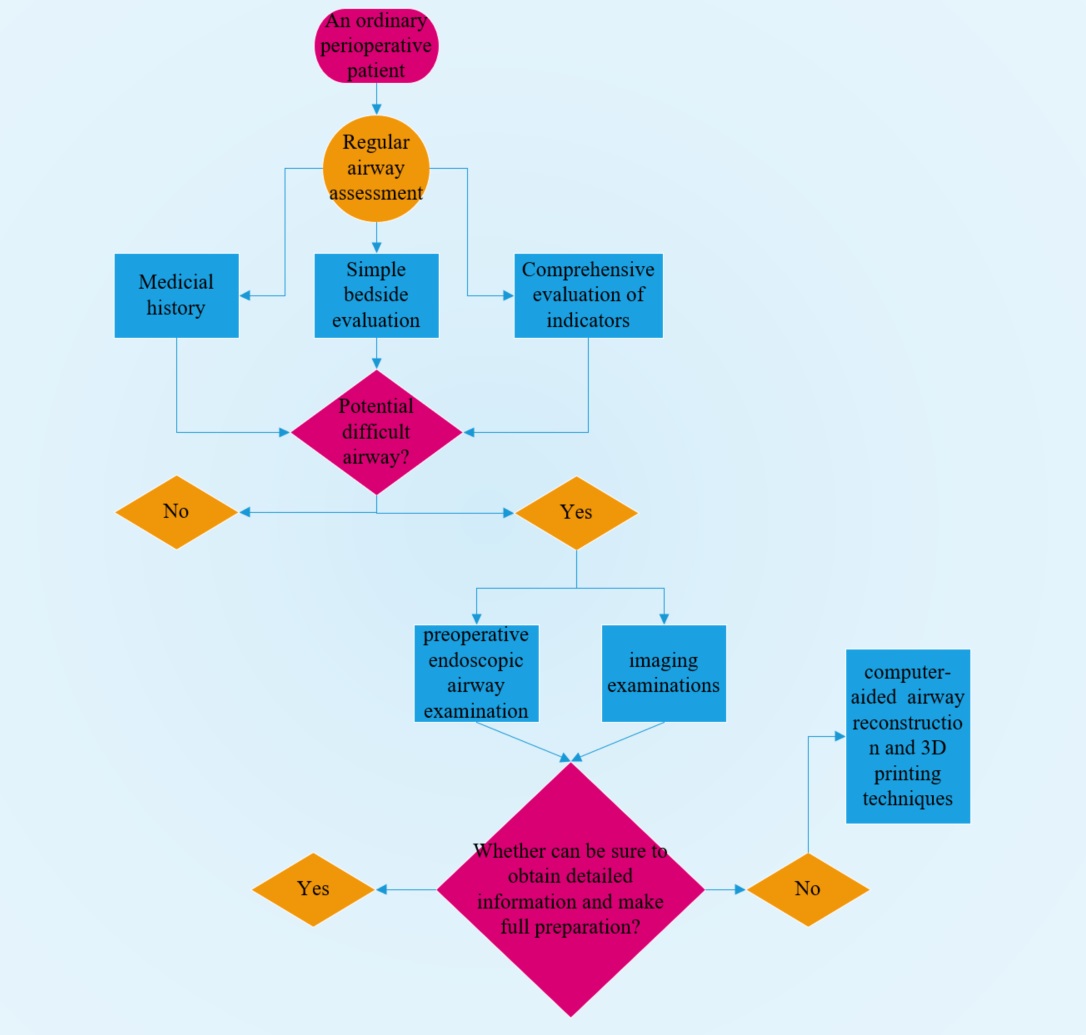


Additional file 1: Figure S3 Flow diagram of determining difficult airway in actual clinical work.

1. Kalezić N, Lakićević M, Miličić B, Stojanović M, Sabljak V, Marković D. Hyomental distance in the different head positions and hyomental distance ratio in predicting difficult intubation. Bosnian journal of basic medical sciences. 2016;16(3):232-6.

2. Cm A, Rao SS, K R, R V, Kn V, T SD. The Ratio of Height to Thyromental Distance (RHTMD) and Height to Sternomental Distance (RHSMD) as the Predictive Tests for Difficult Tracheal Intubation. Cureus. 2022;14(9):e28734.

3. Torres K, Błoński M, Pietrzyk Ł, Piasecka-Twaróg M, Maciejewski R, Torres A. Usefulness and diagnostic value of the NEMA parameter combined with other selected bedside tests for prediction of difficult intubation. Journal of clinical anesthesia. 2017;37:132-5.

4. Siddiqui KM, Hameed F, Ali MA. Diagnostic Accuracy of Combined Mallampati and Wilson Score to Predict Difficult Intubation in Obese Patients: A Descriptive Cross-sectional Study. Anesthesiology and pain medicine. 2021;11(6):e118626.

5. Nørskov AK, Wetterslev J, Rosenstock CV, Afshari A, Astrup G, Jakobsen JC, et al. Effects of using the simplified airway risk index vs usual airway assessment on unanticipated difficult tracheal intubation - a cluster randomized trial with 64,273 participants. British journal of anaesthesia. 2016;116(5):680-9.

6. Ji SM, Moon EJ, Kim TJ, Yi JW, Seo H, Lee BJ. Correlation between modified LEMON score and intubation difficulty in adult trauma patients undergoing emergency surgery. World journal of emergency surgery : WJES. 2018;13:33.

7. Bicalho GP, Bessa RC, Jr., Cruvinel MGC, Carneiro FS, Castilho JB, Castro CHV. A prospective validation and comparison of three multivariate models for prediction of difficult intubation in adult patients. Brazilian journal of anesthesiology (Elsevier). 2023;73(2):153-8.

8. Arné J, Descoins P, Fusciardi J, Ingrand P, Ferrier B, Boudigues D, et al. Preoperative assessment for difficult intubation in general and ENT surgery: predictive value of a clinical multivariate risk index. British journal of anaesthesia. 1998;80(2):140-6.

9. Nausheen F, Niknafs NP, MacLean DJ, Olvera DJ, Wolfe AC, Jr., Pennington TW, et al. The HEAVEN criteria predict laryngoscopic view and intubation success for both direct and video laryngoscopy: a cohort analysis. Scandinavian journal of trauma, resuscitation and emergency medicine. 2019;27(1):50.

10. Acar HV, Yarkan Uysal H, Kaya A, Ceyhan A, Dikmen B. Does the STOP-Bang, an obstructive sleep apnea screening tool, predict difficult intubation? European review for medical and pharmacological sciences. 2014;18(13):1869-74.

11. Ayuso MA, Sala X, Luis M, Carbó JM. Predicting difficult orotracheal intubation in pharyngo-laryngeal disease: preliminary results of a composite index. Canadian journal of anaesthesia = Journal canadien d'anesthesie. 2003;50(1):81-5.
